# Proton Partial Breast Irradiation: Detailed Description of Acute Clinico-Radiologic Effects

**DOI:** 10.3390/cancers10040111

**Published:** 2018-04-07

**Authors:** Valentina Ovalle, Eric A. Strom, Simona Shaitelman, Karen Hoffman, Richard Amos, George Perkins, Welela Tereffe, Benjamin D. Smith, Michael Stauder, Wendy Woodward

**Affiliations:** Department of Radiation Oncology, The University of Texas MD Anderson Cancer Center, Houston, TX 77030, USA; Valeovalle@gmail.com (V.O.); SFShaitelman@mdanderson.org (S.S.); KHoffman1@mdanderson.org (K.H.); r.amos@ucl.ac.uk (R.A.); gperkins@mdanderson.org (G.P.); wtereffe@mdanderson.org (W.T.); BSmith3@mdanderson.org (B.D.S.); MStauder@mdanderson.org (M.S.); wwoodward@mdanderson.org (W.W.)

**Keywords:** breast conservation, breast cancer, proton radiation, proton APBI

## Abstract

*Introduction:* Accelerated partial breast irradiation (APBI) with protons results in a very different acute effect profile than standard whole breast irradiation. We reviewed our initial experience with proton APBI and felt that a detailed description of these effects were needed to permit a common tool to compare experience with this developing technology. *Methods:* Sixty sequential patients treated with proton APBI on a prospective protocol were evaluated and 43 patients with a minimum six-month follow-up underwent detailed photographic and radiologic analysis. The tumorectomy cavity plus an additional 1.5 cm clinical target volume (CTV) was treated with two or three passively-scattered proton beams to a dose of 34 Gy in 10 fractions in one week. Photographs were taken at the end of radiation, at two weeks, six weeks, and every six months thereafter. Mammography was obtained at six months after radiation and annually thereafter. All visual changes were categorized using the smallest meaningful gradations in findings and are demonstrated herein. All treatment-related mammographic findings are reported. *Findings:* Visual and mammographic findings showed a clear time-dependent relationship and significant variation between individuals. Peak skin reaction occurred at two to six weeks after completion of therapy. At two weeks most patients had either no visible effects and patchy erythema involving <50% of the treated skin (60%). At six weeks most patients had either patchy erythema involving <50% of the overlying skin (33%) or patchy erythema involving >50% of the treated skin (28%). Only one patient developed any moist desquamation. At six months most patients had no visible skin changes (57%) or a small, circular area of mild hyperpigmentation (33%). Mammographic changes seen at six months were regional skin thickening (40%), residual seroma (14%), localized retraction (26%), and fat necrosis (2%). A subcategorized variant on the CTCAE 4.0 was developed to foster granular recording of these findings.

## 1. Introduction

According to the US National Cancer Institute database, amongst women with stage 0–II breast cancer treated with breast conserving surgery and radiotherapy from 2003–2010, 10.3% received accelerated partial breast irradiation (APBI), mainly brachytherapy. Utilization of APBI increased from 3.4% in 2003 to 12.4% in 2010 [1]. The potential benefits of APBI are readily apparent when comparing this treatment schedule to longer radiotherapy treatment courses. Accelerated partial breast irradiation is more convenient to patients in terms of time and costs [2]. Most APBI techniques deliver the total radiation dose in a 1–2 week interval, while the more classic whole-breast irradiation schedules take 4–6 weeks [3,4].

There are several techniques to deliver APBI [5]. Amongst the most commonly utilized are brachytherapy, intensity-modulated radiation therapy (IMRT), and 3D-conformal external beam APBI with photons/electrons [1]. More recently proton APBI was introduced with the advantage of being non-invasive while offering high dose conformality and homogeneity.

While clinical experiences with proton APBI with up to five years of follow-up show promising results [6,7], assessments of skin toxicity and cosmesis vary widely across studies. This might be due to the differences in doses, fractionation, number of fields treated per fraction, and scales utilized to measure these endpoints [6,7,8,9]. The groups from Boston and Korea have used a four-point scale to evaluate skin toxicity, ranging from no changes to severe changes [6,8,10], while Bush et al. used the NCICTC criteria [7,11]. (Common Toxicity Criteria, Version 2, of the National Cancer Institute, Rockville, MD, USA)

We considered there was room for further description of evolving acute skin changes and radiologic effects after proton APBI. Our objective was to perform a detailed description of evolving acute skin toxicity in patients with early-stage breast cancer treated with proton APBI and to correlate skin changes following radiotherapy with mammographic findings at six months.

## 2. Patients and Methods

Electronic medical records of patients treated under a prospective phase II trial assessing cosmesis and toxicity of proton APBI at a single institution were retrospectively reviewed. The institutional review board approved this phase II protocol and registered with the NCI. All patients signed patient-specific consent prior to treatment. Patients treated on protocol had to be at least 18 years of age, have a stage 0, I, or II breast cancer with a tumor ≤3 cm, and have ductal carcinoma in-situ (DCIS) or invasive adenocarcinoma on histological examination. Patients with more than three histologically-positive axillary nodes were excluded, as were those with multicentric carcinomas or definitive positive surgical margins. Primary outcomes of this protocol will not be reported herein.

From 2010 to the time of analysis in April 2015, 60 ASTRO consensus suitable/cautionary APBI candidates had been treated with the protocol. Forty-three patients had at least six months of follow-up and, therefore, were included for analysis. Radiotherapy planning details have been published elsewhere [12] and, thus, will be briefly mentioned. The prescribed dose was 34 Gy (RBE) (relative biological effectiveness) in ten fractions, twice daily, over five days using passively-scattered proton beams with a multi-beam technique. We favor the use of three fields in order to spread the skin dose throughout the breast mound, minimizing the area of skin receiving 100% of the dose. The tumor bed inclusive of clips and seroma plus a 15 mm expansion composed the clinical target volume. This volume was then edited to subtract 5 mm from skin and exclude the chest wall. Dose constraints were met in all cases and mirrored NSABP B-39/RTOG 0413 guidelines. It should be noted that there are no accepted planning constraints for skin dose and that most planning systems have known inaccuracies in modeling the dose to the skin.

Clinical evaluations were performed per protocol before and during radiotherapy (RT), then two and six weeks after completion of RT, at six months, and periodically thereafter. Each follow-up visit included medical photographs of the treated breast to assess toxicity and cosmesis. Diagnostic mammograms were performed once a year, starting at the six-month follow-up visit.

### 2.1. Assessment of Skin Changes

**Figure 1:**
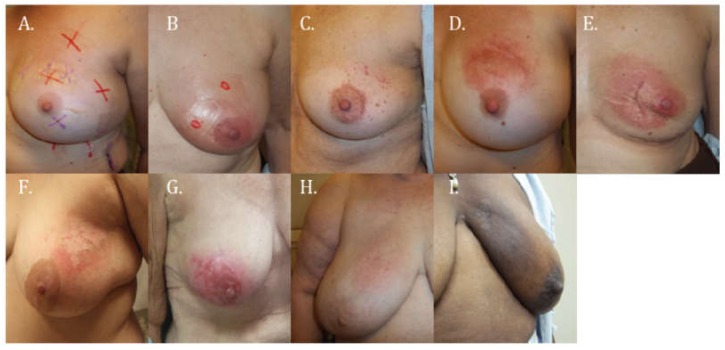
Medical photographs showing examples of different grades of erythema: (**A**) no visual changes; (**B**) faint erythema; (**C**) patchy erythema in ≤50% of the treated skin area; (**D**) patchy erythema in >50% of the treated skin; (**E**) confluent erythema; desquamation; (**F**) dry desquamation; (**G**) moist desquamation; and hyperpigmentation: (**H**) mild hyperpigmentation; and (**I**) moderate/severe hyperpigmentation.

A single physician (VO) reviewed all medical photographs of the 43 patients, taken at the end of RT, at two weeks, six weeks, and six months, and documented all possible skin changes. Using these images and based on subcategorizations of the Common Terminology Criteria for Adverse Events, Version 4 (CTCAE 4.0) , a scale (Table 1) was developed that included all observed skin changes (namely erythema, desquamation, and hyperpigmentation) grading them as follows: (a) erythema: no visual changes, faint erythema, patchy erythema in ≤50% of the treated skin area, patchy erythema in >50% of the treated skin area, or confluent erythema; (b) desquamation: dry or moist; and (c) hyperpigmentation: mild or moderate/severe. Examples of the different grades of erythema, desquamation, and hyperpigmentation included in our skin toxicity scale are shown in Figure 1. All medical photographs were then reviewed again. On that opportunity, the scale was utilized to perform a skin toxicity evaluation for each follow-up visit by assigning a score based on the predominant skin change present. A second physician independently validated this score (ES).

### 2.2. Assessment of Radiologic Changes

Similar to what was done for the skin toxicity evaluation, all six-month follow-up mammograms and their official radiology reports were retrospectively reviewed. After documenting all mammographic findings, a scale was designed (Table 2) incorporating all of these radiologic changes. The scale included four categories evaluating the presence or absence of the following findings: (a) skin thickening; (b) seroma/hematoma; (c) fat necrosis; and (d) retraction/significant asymmetry. Skin thickening was assessed by comparing the index breast to the contralateral breast. If present, the difference in skin thickness was measured in mm. In cases where retraction/significant asymmetry were present, they were subjectively graded as mild, moderate, or severe.

## 3. Results

Forty-three patients with Stage 0–I breast cancer were treated with proton APBI between 2010 and 2014 and had been followed for at least six months. Most patients had a pathologic stage I breast cancer (74.4%) and tumor histology was commonly ductal (79.1%). For invasive disease, the mean tumor size was 8.4 mm (range, 1.6–19 mm) and none had lymphovascular space invasion. All patients received the prescribed 34 Gy (RBE) in ten fractions twice daily according to protocol guidelines. A multi-beam technique with two or three fields was used in 46.5% and 53.5% of the cases, respectively.

### 3.1. Skin Toxicity

**Figure 2:**
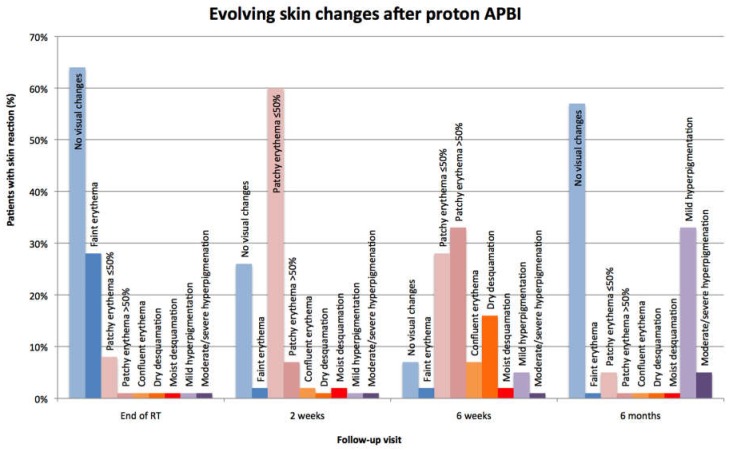
Evolving skin effects of proton accelerated partial breast irradiation (APBI) at the end of radiotherapy, two weeks, six weeks, and six months. APBI, accelerated partial breast irradiation.

Evolving skin toxicity is shown in Figure 2. On physical examination, most patients (64%) had no visual changes on the index breast at the end of radiotherapy. Twenty-eight percent developed faint erythema and only 8% were listed as having patchy erythema in an area ≤50% of the treated skin. At two-weeks, 74% of the patients had developed a visible skin reaction. Changes manifested mainly as patchy erythema in ≤50% of the treated skin area (60%).

**Figure 3:**
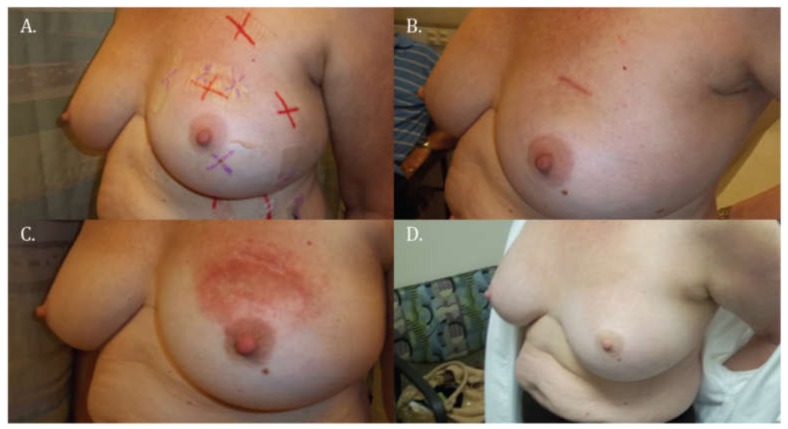
Medical photographs of one patient at selected follow-up visits showing a common skin reaction to proton APBI. No visual changes at (**A**) the end of radiotherapy or at (**B**) the two-week visit. (**C**) Patchy erythema in >50% of the treated skin area at six weeks, which (**D**) resolved showing no visual changes at 6 months. APBI, accelerated partial breast irradiation.

Skin reactions increased from week two to week six of follow-up, when the peak reaction was documented. Almost all patients developed visual skin changes at this time (93%), with patchy erythema in >50% of the treated skin area and ≤50% of the treated skin area in 33% and 28% of all patients, respectively. Sixteen percent of the patients had dry desquamation while moist desquamation was rare (*n*, 1). By six months, most patients had no visual skin changes (57%) and close to one third of the total had developed mild hyperpigmentation in the treated skin (33%). Only occasionally was moderate hyperpigmentation present at this visit (5%). Figure 3 shows the evolving skin reaction to proton APBI in a single patient at different follow-up visits, representing a common scenario.

### 3.2. Radiologic Correlate

**Figure 4:**
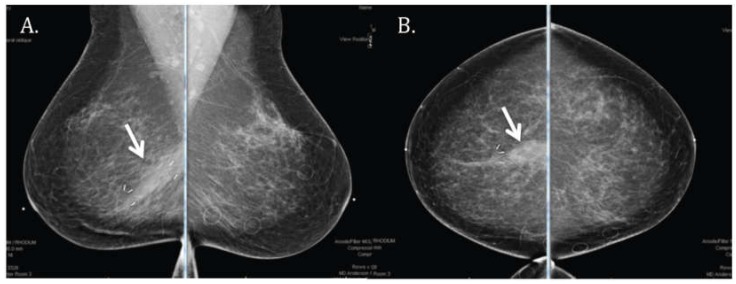
Mammographic evaluation showing (**A**) medio-lateral oblique and (**B**) cranio-caudal views of a common radiologic finding after proton APBI to the right breast at the six-month follow-up visit: post-surgical scar (arrow) and tumor bed clips.

**Figure 5:**
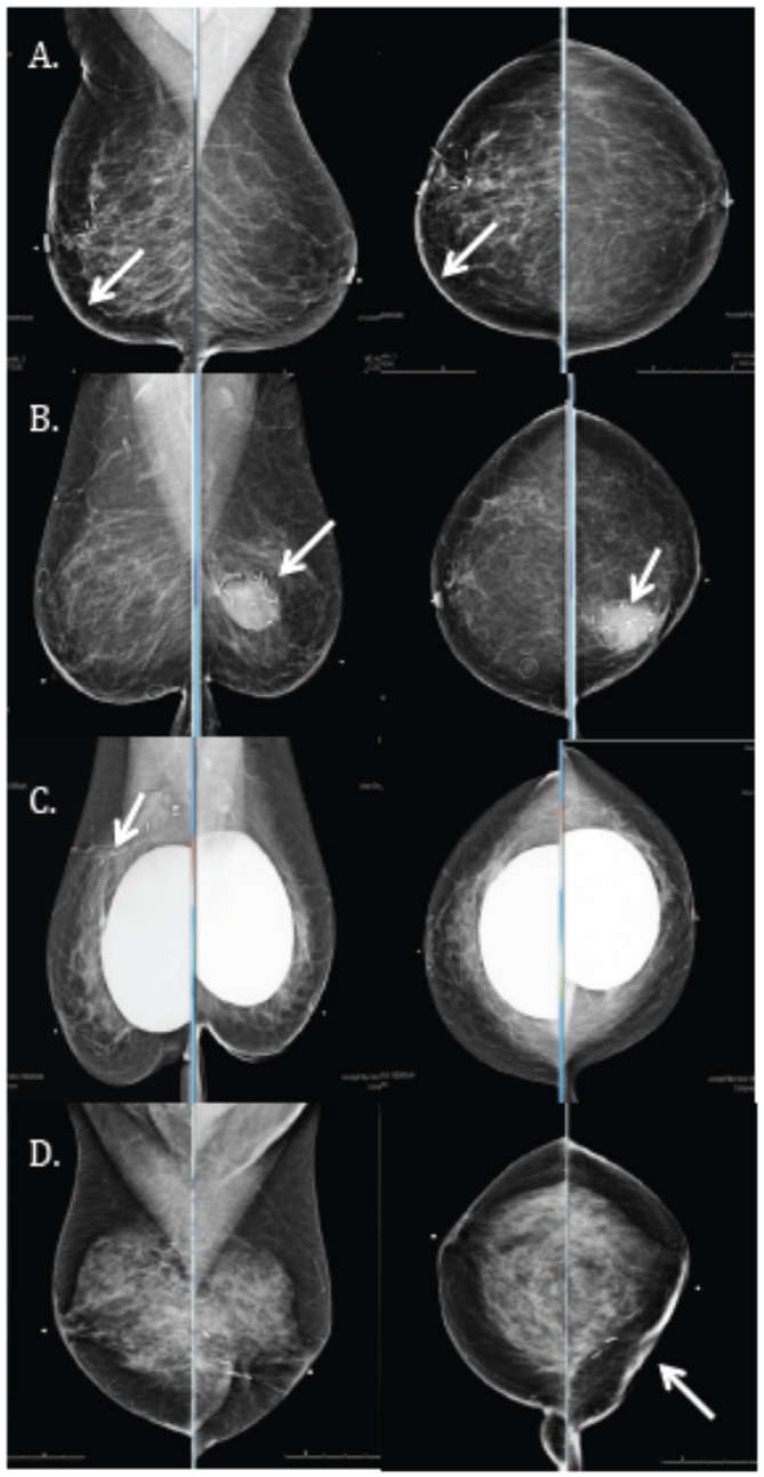
Six-month follow up mammograms showing (arrows) medio-lateral and cranio-caudal views of (**A**) skin thickening; (**B**) seroma/hematoma; (**C**) fat necrosis, and (**D**) retraction/asymmetry.

**Figure 6:**
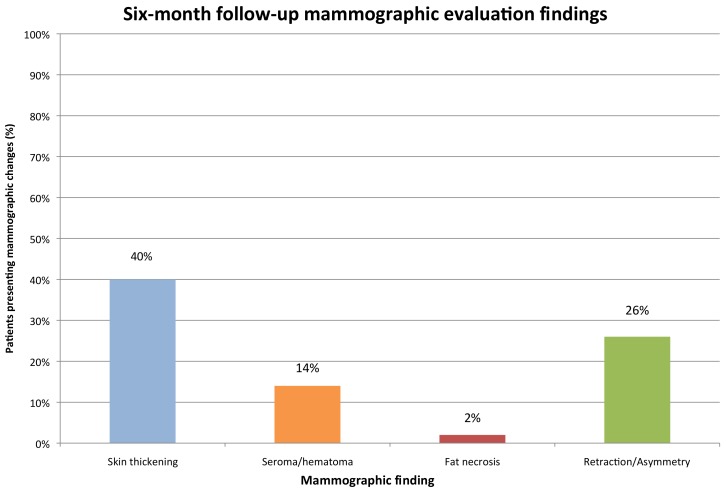
Six-month follow-up mammographic evaluation findings.

The most common finding on the six-month follow-up mammogram (Figure 4) was the presence of a post-surgical scar with tumor bed clips. Examples of other possible findings, such as skin thickening, are shown in Figure 5. An analysis of all mammographic outcomes at six months is shown in Figure 6. Forty percent of the patients had skin thickening at six months. However, this was generally mild, ranging from 0 mm to only 5.5 mm. Retraction/asymmetry was seen in 26% (*n*, 11) of the patients, and graded as mild in 9/11. The presence of a seroma/hematoma was reported in 14% of the cases, while fat necrosis was a rare finding present in only one patient at six months.

## 4. Discussion

Our results suggest that the multi-field proton APBI approach results in moderate early skin reactions only, severe changes being rare, and generally resolves by six months. The same can be said about the six-month follow-up mammographic evaluation, which usually shows only mild post-treatment changes.

Results for skin toxicity evaluations across proton APBI studies have been mixed. Kozak et al. published early results for 20 patients treated on a phase I/II clinical trial with proton APBI [10]. The prescribed dose was 32 Gy (RBE) using 4 Gy (RBE) fractions twice daily over four days. For patients treated with a multi-beam technique, only one field was treated per fraction. Importantly, skin toxicity was graded with a four-point scale ranging from 0 (none) to 3 (severe). According to physician assessment at the 3–4 week follow-up visit 58% and 21% of the patients had moderate and severe skin color changes, respectively. At 6–8 weeks severe changes increased to 37% of the patients, while moderate changes decreased to 16%. By six months, moderate skin color changes were still seen in 28% and mild changes in 44% of the patients.

Chang et al. treated 30 patients using a single- or two-field proton APBI beam technique with 30 Gy (RBE) in 6 Gy (RBE) fractions, once daily, over five days [8]. Using the same scale as the Boston group, they reported fewer skin toxicity. Erythema/hyperpigmentation was mild in 70% of the patients and the end of RT, while at two months it was moderate in 30% and mild in 70%. At six months, moderate changes had decreased to 13% and were mild in 80%. No severe erythema or hyperpigmentation were reported. Wet desquamation peaked at two months and was mild in 13% of the patients, moderate in 3%, and severe in 3% at that follow-up visit.

The group from Loma Linda University first published their results in 2011 [13]. Using NCICTC v.2.0 (Common Toxicity Criteria, version 2, National Cancer Institute), they assessed toxicity in 50 patients treated with proton APBI using a multi-beam technique with 4 Gy (RBE) fractions once daily, over ten days. Acute skin toxicity was reported as grade 1 in 26/50 patients and grade 2 in 4/50. No patients developed ≥ grade 3 acute skin toxicity. 

These studies seem to have conflicting toxicity results, especially when the very mild acute toxicity of the group from Loma Linda is contrasted against the experience from Boston. Thus, what differences in treatment could account for such contrasts? First, the diverse treatment schedules selected were likely to contribute. As we have previously mentioned [11], though the total dose used by Bush et al. was higher than the one utilized by the Boston group, the biologic intensity and shorter overall treatment time used by the latter group might have contributed to their higher toxicity. Second, all three groups used different approaches to treatment planning. Better results were seen when a multi-field technique was used as compared to single fields [8], and probably when all planned fields were treated per fraction as opposed to treating only one field. Additionally, one could argue that the improved toxicity profile reported by Bush et al. is, in part, due to smaller expansions utilized for their planning target volumes (PTVs) when compared to the Boston group. All of these published experiences have used passively-scattered protons. While scanned-beam protons may be able to further improve the skin dose, the trade-off with planning uncertainty remains a concern especially for shallow tumors [9]. To date only 25 breast patients have been reported as having received scanned-beam protons, and this was in the post-mastectomy setting [10].

Another relevant component to consider is how toxicity was measured. The groups from Boston and Korea used a very rigorous and sensitive scoring system capable of discerning between cutaneous effects involving 0, 1–2 cm^2^, 2–4 cm^2^, and >4 cm^2^. In contrast, the NCICTC v2.0 used at Loma Linda University is rather ambiguous, including several different skin reactions in one single grade category. For example, Grade 2 radiation dermatitis includes moderate to brisk erythema and patchy moist desquamation. Dissimilarities across toxicity scales are evident, and make comparisons between studies very challenging. 

As patients in our phase II trial [14] were being seen for follow-up, we realized that a toxicity scale designed exclusively for proton APBI that could provide more granularity was warranted. Since published clinical experiences with proton APBI are limited, but are likely to expand shortly, we decided to create a toxicity scale that would be detailed in terms of description, but also broad in terms of including all possible skin effects. We tried to accomplish this by including all skin changes seen on medical photographs at different follow-up visits in the scale’s design and then assigned these findings to a subcategorized variant of the CTCAE 4.0 [15].

The defined follow-up timeline stated in a protocol will determine whether evolving skin changes are observed before, during, or after their peak reaction. This is another confounding factor when comparing skin toxicity across studies. Per protocol, patients in our study were seen at the end of RT, at two weeks, six weeks, and six months. Skin toxicity increased between follow-ups at weeks two and six, finding a peak skin reaction at six weeks, and generally resolved by six months. From our experience and those previously published, one can infer that the peak skin reactions generally present between the 4th and 8th week after RT. In our cohort, skin color changes at six months almost halved those published by Kozak et al. and Chang et al. [8,12], while at six weeks they were rather similar. Importantly, only 2% of the patients presented wet desquamation at six weeks, while this ranged from 20–22% in previous studies. The lower probability of skin changes seen herein might be explained by our treatment schedule and technique, especially considering the lower dose per fraction (3.4 Gy (RBE)) utilized and the fact that all treatments were planned with multiple proton beams treated on every fraction [14]. 

In our experience, the multi-field proton APBI approach results in moderate skin reactions only and severe changes are rare. Mammographic changes at six months are mild and may include skin thickening, seroma/hematoma, mild retraction/asymmetry and, rarely, fat necrosis. Though the toxicity scale utilized herein was created specifically for the assessment of proton APBI and needs validation, it appears to be a more granular method of measuring and describing in detail skin changes occurring after proton APBI. The use of a single skin toxicity scale across institutions, similar to the one we have proposed, would facilitate comparing outcomes amongst patients treated with this emerging modality.

## 5. Conclusions

The acute skin and radiologic toxicity of proton APBI using a supine multibeam technique and passively-scattered protons has a very favorable profile. While further time is required to assess the clinical effectiveness and long-term toxicity of this approach, the advantages of a five-day treatment using a highly-conformed radiation beam which requires neither indwelling catheters nor delivers any dose to non-target organs should be further explored. A subcategorized scoring system of the CTCAE 4.0, with temporal correlations is included herein so that those centers beginning to deploy this treatment for breast cancer can easily assess their outcomes using a technique-specific tool.

## Figures and Tables

**Table 1:** Skin toxicity scale.

Main Skin Reaction	CTCAE 4.0 Grade (Subcategorized)	Description
Erythema	0	No visual Changes
	1(a)	Faint erythema
	1(b)	Patchy erythema in ≤50% of the treated skin area
	1(c)	Patchy erythema in >50% of the treated skin area
	2(a)	Confluent erythema over entire treated area
Desquamation	1(d)	Dry desquamation limited to treated area
	3	Moist desquamation limited to treated area not in skin folds
Hyperpigmentation	1(a)	Mild hyperpigmentation limited to treated area
	1(b)	Moderate/severe hyperpigmentation limited to treated area

**Table 2:** Mammographic findings at the six-month follow-up visit.

Mammographic Finding	CTCAE 4.0 Grade
Skin thickening	NA
Seroma/hematoma	1
Fat necrosis	1
Retraction/Asymmetry	Mild (1)
	Moderate (2)
	Severe (3)
